# Serum levels of biomarkers that may link chronic obstructive pulmonary disease and depressive disorder

**DOI:** 10.1007/s43440-023-00548-3

**Published:** 2023-11-03

**Authors:** Elżbieta Małujło-Balcerska, Tadeusz Pietras, Witold Śmigielski

**Affiliations:** 1 2nd Chair of Internal Diseases, Department of Pneumology, Medical University of Łódź, 22Nd Kopcińskiego Street, 90-153 Lodz, Poland; 2 Department of Clinical Pharmacology, Medical University of Łódź, Lodz, Poland; 3 Second Department of Psychiatry, Institute of Psychiatry and Neurology, Warsaw, Poland; 4 Department of Epidemiology, Cardiovascular Disease Prevention and Health Promotion, The Cardinal Stefan Wyszynski National Institute of Cardiology, Warsaw, Poland

**Keywords:** Inflammation, Chronic obstructive pulmonary disease, Depressive disorder

## Abstract

**Background:**

Depressive disorder is a common comorbidity of chronic obstructive pulmonary disease (COPD); according to some studies, it occurs in approximately 80% of patients. The presence of depressive symptoms influences the quality of life and affects the course and treatment of this disease. The cause of depressive symptoms in COPD and the linking mechanism between COPD and depressive disorder have not been clearly elucidated, and more studies are warranted. Inflammation and inflammation-related processes and biomarkers are involved in the etiology of COPD and depressive disorder and may be an explanation for the potential occurrence of depressive disorder in patients diagnosed with COPD. The scope of this study was to measure and compare the profiles of IL-18, TGF-β, RANTES, ICAM-1, and uPAR among stable COPD patients, recurrent depressive disorder (rDD) patients, and healthy controls.

**Methods:**

Inflammation and inflammation-related factors were evaluated in COPD patients, patients diagnosed with depressive disorder, and control individuals using enzyme-linked immunosorbent assays.

**Results:**

Interleukin (IL)-18, transforming growth factor (TGF)-β, chemokine RANTES, and urokinase plasminogen activator receptor (uPAR) concentrations were higher in patients suffering from COPD and depression than in control patients. Intercellular adhesive molecule (ICAM)-1 levels were significantly higher in COPD patients and lower in depressive disorder patients than in controls.

**Conclusions:**

Higher levels of IL-18, TGF-β, RANTES, and uPAR in patients with COPD might indicate the presence of depressive disorder and suggest the need for further evaluation of the mental state of these patients.

## Introduction

Chronic obstructive pulmonary disease (COPD) is a common, widely diagnosed disease [1]. The role of inflammation in COPD etiology is well explained, involving high levels of inflammatory mediators, including cytokines and chemokines, and interactions with other process-related biomarkers. The inflammatory process influences the remodeling of the lower airways and is associated with well-documented elevations of some cytokines, including tumor necrosis factor (TNF)-α, interferon (IFN)-ɣ, interleukin (IL)-4, and IL-10 [2]. High levels of cytokines, such as IL-1, IL-6, TNF-ɑ, IFN-ɣ, IL-4, and IL-10, have also been found to be biomarkers of depressive disorder [3]. These cytokines can be hazard agents for and potentiate the possibility of the occurrence of depressive mood and general depressive disorder in COPD patients. Other candidate lung and peripheral systemic biomarkers of COPD include IL-18, transforming growth factor (TGF)-β, the chemokine RANTES, intercellular adhesive molecule (ICAM)-1, and urokinase plasminogen activator receptor (uPAR). These biomarkers are known to be related to the clinical outcome of lung function and are positive and/or negative biomarkers reflecting COPD severity. An association between a high concentration of IL-18 in the serum of COPD patients and a decline in lung function parameters allows IL-18 levels to be used as a marker of the degree of impaired airway obstruction in COPD [4]. Serum TGF-β levels have been found to be higher in COPD patients than in controls, whereas the highest levels were found in patients with Global Initiative for Chronic Obstructive Lung Disease (GOLD) stage 4 according to spirometry, suggesting a stage-dependent association between systemic TGF-β levels and lung function [5]. In addition, TGF-β correlates with severity and airflow limitation in COPD [6]. The bronchial epithelial expression of the TGF-β protein correlates with the number of macrophages recruited into the airway epithelium in COPD [7]. In a cross-sectional study, serum RANTES concentrations were higher in patients diagnosed with COPD than in control patients [8]. The increased expression of RANTES in the bronchial mucosa of patients with stable COPD and the release of RANTES by lung tissue suggest its involvement in the disease [9, 10]. Measurement of the serum concentration of ICAM-1 in COPD patients has provided strong support for the involvement of the systemic component in COPD [11]. Blocking ICAM-1 signaling prevents viral adhesion and COPD exacerbation [12]. In addition, expression of the gene encoding ICAM-1 is upregulated in the fibroblasts of COPD patients [13]. Compared to IL-18, TGF-β, RANTES, and ICAM-1, less is known about the role of uPAR in COPD. However, the relationship between the uPAR system, lung function, small airway fibrosis, and emphysema suggests a role in COPD [14]. Peripheral uPAR levels are elevated in COPD and are related to arterial stiffness [15]. uPAR, as a novel biomarker, has potential value in the early diagnosis of COPD and the prediction of acute exacerbation (AE) COPD [16].

Similar to COPD, IL-18, TGF-β, RANTES, ICAM-1, and uPAR have been explored in depressive disorder. IL-18 serum levels have been found to be higher in major depressive disorder patients than in controls [17]. Peripheral IL-18 correlates with abnormal brain activity in patients with depression, suggesting that IL-18 is involved in the pathophysiological mechanism of depression [18]. A relationship between IL-18 and opioid neurotransmission in response to induced sadness has been identified, suggesting its involvement in signaling pathways in depressed individuals [19]. Analysis of the serum levels of TGF-β observed significant increase of this molecule in patients with depressive disorder comparing to controls [20]. A preclinical study by Białek et al. [21] confirmed an association between TGF-β gene expression and methylation in depressive disorder. In addition, antidepressant treatment reduces higher TGF-beta levels to within the normal range [22]. According to our previous study, RANTES concentration was higher in depressive patients than in controls [23]. Serum RANTES levels have been found to be reduced after treatment [24]. Upregulation of RANTES within brain tissue was found in adult female mice with depressive-like behavior [25]. The upregulation of adhesion molecules, including ICAM-1, and the possible role of vascular pathology in depression development have been studied. Receiver operating characteristic (ROC) curve analysis demonstrated that a combined panel of IL-1α, IL-5, and ICAM-1 achieved high accuracy in discriminating antidepressant non-responders from responders (area under the curve (AUC) = 0.850, sensitivity = 83.3%, specificity = 81.8%) [26]. ICAM-1 has also been found to be a predictor of depression risk following traumatic brain injury [27]. Higher peripheral levels of uPAR were observed by Małujło-Balcerska et al. [28] in patients with major depressive disorders. A significantly higher risk for frequent antidepressant use in patients with higher uPAR values was also observed by Haastrup et al. [29].

The molecules described above may interact with each other and interfere with different processes. The involvement of IL-18, TGF-β, and RANTES in COPD has been observed, with these molecules interfering with vascular network factors that disturbances are characteristically observed in COPD [30]. TGF-beta causes an increase in endothelial ICAM-1 expression and lung injury [31]. The link between cytokines and fibrinolytic system factors, including uPAR, initiates inflammatory responses in macrophages [32]. In addition, uPAR is increased during inflammation and participates in vascular disturbances, such as atherosclerosis [15]. Vascular effects of cytokines have been described, and accumulating evidence suggests a role for cytokines in vascular dysfunction [33]. The cytokine ICAM-1, as an adhesion molecule, may be involved in the depression pathomechanism according to the vascular hypothesis of depressive disorder [34]. The link between uPAR and depression development and vascular dysfunction has also been described [35]. Circulating cytokines and other biomarkers are able to cross the blood–brain barrier and may cause vascular dysfunction [34], one of the processes related to depressive disorders, especially late-life depression [36].

COPD influences patient quality of life, and disorder in mental health, such a presence of depressive disorder and inflammation, may importantly participate in reducing quality of life for COPD patients [37]. The presence of high depressive symptoms at baseline was associated with subsequent moderate-to-severe exacerbations and hospital admissions in patients with COPD [38], while diagnosis and antidepressant therapy reduced depressive symptoms in all stages of COPD and improved quality of life [39].

Considering the frequency of diagnosis of depression during COPD and the fact that depressive disorder may be undiagnosed or masked, explanation is needed for biomarkers and pathways that join COPD and depressive disorder. Thus, the presence of some parameters in the periphery may establish chance for the occurrence of depression in COPD patients.

In this study, we evaluated the concentrations of inflammation and vascular process-associated factors in patients with COPD, patients with depressive disorder, and control patients with the aim to compare the profile of examined biomarkers between COPD and depressive disorder and to find if these diseases may share a biological mechanism. In addition, we aimed to discuss if these molecules might predict the possibility of depressive symptoms in COPD patients. We examined and confronted the ranges of IL-18, TGF-β, RANTES, ICAM-1, and uPAR among stable COPD patients, recurrent depressive disorder (rDD) patients, and healthy controls.

## Materials and methods

A group of twenty-five patients (11 women, 14 men) with stable COPD [age: mean (SD), 61.7 (6.82); median (IQR), 61 (57–67)], twenty-five patients (10 women and 15 men) with rDD [age: mean (SD), 61.22 (5.88); median (IQR), 61 (58–66)], and twenty-five (9 women, 16 men) healthy controls [age: mean (SD), 61.16 (6.74); median (IQR), 61 (57–67)] took part in this study. The enrolled groups were age and gender matched (statistical analysis for age: *H* = 0.01, *p* = 0.99 and sex:* χ*^*2*^ = 0.33, *p* = 0.85, similar to as described in our previous study [40]). Diagnosis of both COPD and rDD was based on the ICD-10 criteria [41]. Diagnosis of COPD was based on the GOLD standard criterion of a post-bronchodilator ratio of forced expiratory volume in 1 s (FEV_1_) to forced vital capacity (FVC) < 0.70. [42] The FEV1/FVC values in COPD patients were 61% (56–68). The inclusion criteria were a diagnosis of COPD according to GOLD and age > 40, and healthy controls were matched for age and sex. The exclusion criteria were as follows: COPD exacerbation 8 weeks prior to sample collection, pulmonary disease other than COPD, systemic inflammatory diseases, and infection. The inclusion process was carried out by a qualified psychiatrist. The 17-Hamilton Depression Rating Scale (HAM-D) was used to assess depressive disorder severity [43]. The HAM-D values in rDD patients were 21 (15–27). The exclusion criteria were as follows: psychiatric diseases other than depressive disorder, immunological diseases, acute or chronic infection, substance abuse, and treatment with anti-inflammatory agents. All procedures were reviewed and approved by the local bioethics committee. Written informed consent was obtained from all the participants of this study. All patients and control subjects who participated in this study were unrelated and native inhabitants of central Poland.

IL-18, TGF-β, RANTES, soluble ICAM-1, and soluble uPAR were measured in serum using enzyme-linked immunosorbent assay (ELISA) kits (R&D Systems, Inc., MIN USA). All calculations were performed according to the instructions provided by the manufacturers.

The ELISA results are expressed as the median (M) interquartile range (IQR). Since the data did not follow normal distribution, the Kruskal‒Wallis test followed by the Dunn’s post hoc test and Spearman`s rank correlation coefficient were calculated to identify differences or correlations. Differences were considered significant if the p-value was 0.05 or less.

## Results

**Fig. 1:**
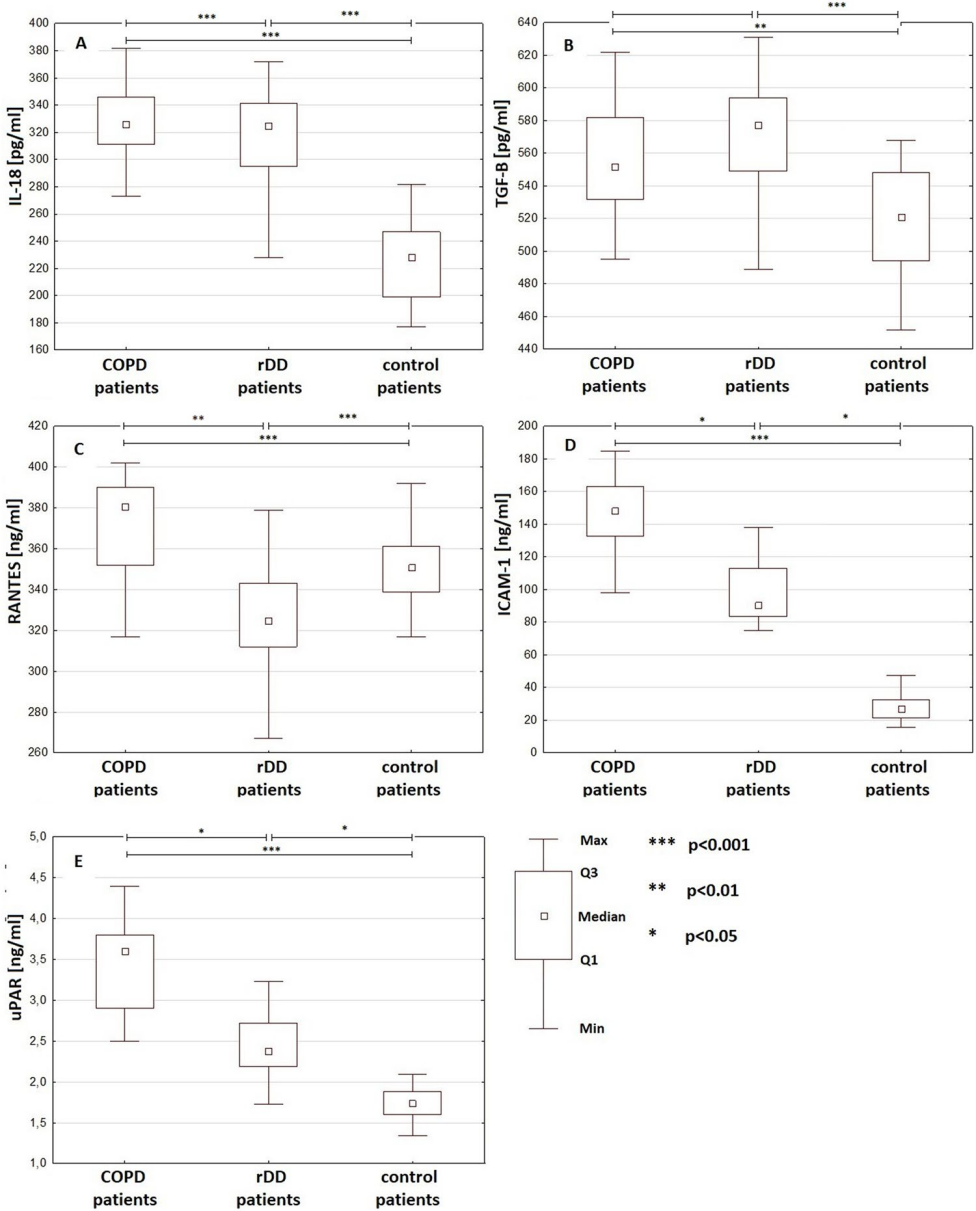
Comparison of serum levels of IL-18, TFG-β, RANTES, ICAM-1, and uPAR in patients with chronic obstructive disorder (COPD), recurrent depressive disorder (rDD), and control group participants. Statistical analysis, the Kruskal‒Wallis test followed by the Dunn’s post hoc. Boxes represent the median and the first quartile (Q1)—the third quartile (Q3) range, and whiskers represent the minimum (min.)–maximum (max.) value. IL-interleukin, *TGF* transforming growth factor, *ICAM* intercellular adhesive molecule, *uPAR* urokinase plasminogen activator receptor

Detailed data are presented in Table 1 and Fig. 1

**Table 1:** Comparison of serum levels of IL-18, TFG-β, RANTES, ICAM-1, uPAR in patients with chronic obstructive disorder (COPD), recurrent depressive disorder (rDD), and control group participants

	COPD patients, *n* = 25	rDD patients, *n* = 25	Control patients, *n* = 25	*p*-value: COPD vs. controls, rDD vs. controls, COPD vs. rDD
IL-18 (pg/ml)	326 (311–346)	325 (295–431)	228 (199–247)	*p* < 0.0001; *p* < 0.0001; *p* > 0.05
TGF-β (pg/ml)	552 (532–582)	577 (549–594)	521 (494–548)	*p* = 0.0005; *p* < 0.0001; *p* = 0.72
RANTES (ng/ml)	148 (133–163)	90.38 (83.43–113.20)	26.87 (21.51–32.30)	*p* < 0.0001; *p* < 0.0001; *p* = 0.006
ICAM-1 (ng/ml)	381 (352–390)	325 (312–343)	351 (339–361)	*p* = 0.038; *p* = 0.039; *p* < 0.0001
uPAR (ng/ml)	3.60 (2.9–3.8)	2.38 (2.19–2.72)	1.74 (1.6–1.88)	*p* < 0.0001; *p* < 0.0001; *p* < 0.006

Data are expressed as medians and interquartile ranges (IQR)

Statistical analysis: the Kruskal‒Wallis test followed by the Dunn’s post hoc test, *n* number of participants IL-interleukin, *TFG* transforming growth factor, *ICAM* intercellular adhesive molecule, *uPAR* urokinase plasminogen activator receptor

### IL-18 levels in serum of COPD patients, rDD patients, and control patients

IL-18 serum concentrations (pg/ml) measured with ELISA method were significantly higher in patients with COPD and in patients with rDD than those in control patients (Kruskal–Wallis test, *H* = 46.11, *p* < 0.0001).

### TGF-β levels in serum of COPD patients, rDD patients, and control patients

TGF-β serum concentrations (pg/ml) measured with EISA method were significantly higher in patients diagnosed with COPD and in patients diagnosed with rDD than those in control patients (p= 0.005 and < 0.0001, respectively). There were no significant differences between the COPD and rDD groups (Kruskal–Wallis test, *H* = 19.43, *p* = 0.72).

### RANTES levels in serum of COPD patients, rDD patients, and control patients

RANTES serum concentrations (ng/ml) measured with EISA method were significantly higher in patients with COPD and in patients with rDD than those in control patients (p < 0.0001). There was also a significant difference between the COPD and rDD groups (Kruskal–Wallis test, *H* = 58.53, *p* = 0.006).

### ICAM-1 levels in serum of COPD patients, rDD patients, and control patients

ICAM-1 serum concentrations (ng/ml) measured with EISA method in COPD patients were higher than those in rDD patients (*p* = 0.0001) and in healthy controls (*p* = 0.038). ICAM-1 levels in rDD patients were significantly lower than those in healthy controls (Kruskal–Wallis test, *H* = 24.61, *p* = 0.039).

### uPAR levels in serum of COPD patients, rDD patients, and control patients

uPAR serum concentrations (ng/ml) measured with EISA method were significantly higher in patients with COPD and rDD than those in healthy controls (*p* < 0.0001). There was also a significant difference between the COPD and rDD groups (Kruskal–Wallis test, *H* = 55.90, *p* = 0.006).

## Discussion

To our knowledge, there are a few published studies comparing the profile of biomarkers between COPD patients and those with depressive disorder. In the present study, not only similarities but also different results in serum concentrations of biomarkers in both disorders were observed. Results of this study yielded new data on inflammation-associated factors participating in appearance of depression during COPD. Obtained data suggest biomarkers that may determine the occurrence of depression in COPD patients.

IL-18 is known as an important cytokine with numerous and diverse roles. In our study, we found that the levels of IL-18 in COPD and rDD patients were very similar. This suggests that the IL-18-associated mechanisms is similar in both diseases and that IL-18 can predict the occurrence of depressive symptoms in COPD patients. IL-18 is released from pro-IL-18 by caspase-1-related mechanism within the NLRP3 inflammasome. In the induction of the NLRP3 inflammasome pathogen-activated molecular patterns (PAMPs) and damage-activated molecular patterns (DAMPs) play a role [44]. These molecules are known to have a role in COPD development [45] and may also play a role in depression. Regarding the similarity in the concentrations of IL-18 in COPD and rDD, it can be suggested that there are not only no differences between systemic levels but also no differences among the lung and brain areas. The recently described both axes gut–lung and gut–brain can affect inflammation, as the microbiome may influence the NLRP3 inflammasome reaction and levels of L-18 expression [46]. TGF-β regulates a plethora of biological processes and may contribute to a broad range of pathologies [47]. Similarly, the profile of TGF-β did not differ between both, patients with COPD and patients with rDD, which suggests that TGF-β is a molecule linking COPD and depressive disorder. One of the mechanisms that can increase the expression of TGF-β in both diseases is oxidative stress; reactive oxygen species that are characteristically found in COPD and rDD pathways [48, 49] may induce TGF-β expression levels, while active TGF-β may induce a redox imbalance and other inflammatory-related molecules through nuclear factor (NF)-κB [50]. Although in the case of TGF-β, the percentage differences between values for COPD in comparison to the control group (6.0% higher) or for rDD in comparison to the control group (10.7% higher) are not so high, the statistical analysis found that observed differences are statistically significant. It needs further studies to confirm and explain if the differences may have useful importance. RANTES is another inflammation-related molecule that can link COPD and depressive disorder and/or can explain the probable presence of depression in COPD patients. One of the roles played by RANTES in the development of depressive symptoms in COPD patients may be similar to that of RANTES in other chemokine-derived vascular diseases, which are known causes of depression development [34]. ICAM-1 is a master regulator of many essential functions and ICAM-1 expression is highly induced by inflammatory stimuli [51]. In our study, higher values of ICAM-1 in COPD confirmed the inflammation-based mechanism of this disease. However, the lower concentration of ICAM-1 in depressive patients is not consistent with the inflammatory and ICAM-related vascular hypothesis of depressive disorder [34]. According to the results of our study, ICAM-1 cannot be considered one of the elements linking depressive disorder and COPD. Adhesion molecules may be involved in different pathways of these diseases. For example, in some groups of depressed patients, lower ICAM-1 levels may reduce the risk of coronary disease [52]. The results of our study are consistent with evidence that uPAR may be upregulated in COPD [15] and depressive disorder [28]. Our study suggests that uPAR is a linking molecule between COPD and rDD because the levels of uPAR are higher in patients with both diseases than in controls. uPAR, the peripheral levels of which are related to arterial stiffness [15], may be one of the factors linking inflammation and vascular dysfunction. These results should be further investigated with other fibrinolytic and inflammation system biomarkers as potential biomarkers for predicting the development of depressive symptoms in COPD.

The occurrence of depressive disorder in patients with COPD may be a consequence of direct inflammation-associated mechanism and their possible interference with other processes, i.e., vascular processes. Considering our study, the vascular theory of depression [34], the role of cytokines in vascular disturbances [33], and increased inflammatory molecules may explain the possible mechanism of depressive symptoms in COPD through inflammation-associated vascular abnormalities, especially in late-life depression. Disturbances in the vascular network may appear in the periphery, lungs, and brain.

The identification of systemic biomarkers that may link COPD and depressive disorder may guide future therapeutic interventions, such as early intervention in assessing and monitoring mental health in COPD patients and early psychological and/or pharmacological therapeutic intervention. This could reduce and/or prevent the development of depressive symptoms in high-risk COPD patients. Before therapeutic intervention in patients at risk of developing depressive symptoms, the use of omega-3 polyunsaturated fatty acids, which play a role in regulating inflammation, may be discussed [53]. A reduction in depressive symptoms results in a better quality of life and better control of COPD. In addition, integrated telehealth care for COPD patients with depression can reduce emergency department visits [54].

### Limitations

Limitations to this study include the following: first, the small sample size might have resulted in sampling bias. Second, patients enrolled in this study were treated with medicines for COPD symptoms and depressive disorder symptoms, which may influence the results. Additionally, a group of COPD patients with depressive symptoms self-assessed by Beck's inventory or COPD patients diagnosed with the depressive disorder would add value to this study.

## Conclusions

COPD patients and depressive disorder patients present similar profiles of inflammatory biomarkers, such as IL-18, TGF-β, and RANTES, and interact with the inflammation, biomarker of the fibrinolytic system, uPAR. These molecules may be evidence of shared biological mechanisms between investigated diseases and are supposed to be putative agents in the mechanism of the occurrence of depressive disorder in patients with COPD. Patients with COPD with high levels of IL-18, TGF-β, RANTES, and uPAR compared to healthy controls warrant examination for the appearance of depressive symptoms and therapeutic intervention for the prevention of depressive symptoms.

## Data Availability

The data analyzed during this study are available from the corresponding author upon reasonable request.
